# TB antigen-based skin tests and QFT-Plus for *Mycobacterium tuberculosis* infection diagnosis in Brazilian healthcare workers: a cost-effectiveness analysis

**DOI:** 10.1590/0102-311XEN178623

**Published:** 2025-02-24

**Authors:** Fernanda Mattos de Souza, Ricardo E. Steffen, Márcia Ferreira Teixeira Pinto, Thiago Nascimento do Prado, Ethel Leonor Noia Maciel, Anete Trajman

**Affiliations:** 1 Universidade do Estado do Rio de Janeiro, Rio de Janeiro, Brasil.; 2 Centro Biomédico, Universidade do Estado do Rio de Janeiro, Rio de Janeiro, Brasil.; 3 Instituto Nacional de Saúde da Mulher, da Criança e do Adolescente Fernandes Figueira, Fundação Oswaldo Cruz, Rio de Janeiro, Brasil.; 4 Programa de Pós-graduação em Saúde Coletiva, Universidade Federal do Espírito Santo, Vitória, Brasil.; 5 Centro Biomédico, Universidade Federal do Espírito Santo, Vitória, Brasil.; 6 McGill University, Montreal, Canada.; 7 Universidade Federal do Rio de Janeiro, Rio de Janeiro, Brasil.

**Keywords:** Healthcare Workers, *Mycobacterium tuberculosis* Infections, Diagnosis, Cost-Effectiveness Analysis, Trabalhadores da Saúde, Infecção por *Mycobacterium tuberculosis*, Diagnóstico, Análise de Custo-Efetividade, Trabajadores de la Salud, Infección por *Mycobacterium tuberculosis*, Diagnóstico, Análisis de Costo-Efectividad

## Abstract

This study aimed to analyze the cost-effectiveness of three tuberculosis (TB) antigen-based skin tests (TBST) (Diaskintest, C-TST, and Cy-TB) and QFT-Plus for TB infection diagnosis compared to the current standard of care, PPD Rt-23 tuberculin skin test (TST), among healthcare workers in Brazil. A state-transition Markov model was employed, simulating a cohort of healthcare workers (five annual cycles) for testing and treating TB infection with three months of weekly doses of rifapentine and isoniazid (3HP) under the Brazilian public health system perspective. Effects (TB disease averted) and costs for screening and treating TB infection were discounted at 5%. Incremental cost-effectiveness per TB averted was estimated. One-way and probabilistic sensitivity analysis were performed. Brazil, an upper-middle-income country with a high burden of TB, shows one of the largest universal public health systems and provides free-of-charge diagnosis and treatment for TB and TB infection. TST is the standard of care, whereas QFT-Plus is available for very high-risk populations. The three new TBST are under validation for eventual incorporation. Patients or participants: a hypothetical cohort of 10,000 healthcare workers, working at any level of healthcare service, and negative TST results in the previous year of both sexes with a baseline negative TST result. Diaskintest, C-TST, Cy-TB, and QFT-Plus were found to show a higher specificity. Costs with QFT-Plus were higher due to equipment, human labor, and test price. Diaskintest was the most cost-saving strategy, followed by Cy-TB for TB preventive treatment with 3HP. In the Brazilian scenario, Diaskintest and Cy-TB are the most cost-effective tests for sequential testing of healthcare workers.

## Abstract (pt)

Este estudo objetivou analisar o custo-efetividade de três testes cutâneos baseados em antígenos de tuberculose (TB) (Diaskintest, C-TST, Cy-TB) e QFT-Plus para diagnóstico de infecção por TB comparando-os ao padrão de atendimento atual, PPD Rt-23 teste tuberculínico (TT), entre profissionais de saúde no Brasil. Trata-se de um modelo de Markov de transição de estado, simulando uma coorte de profissionais de saúde (cinco ciclos anuais) para testagem e tratamento de infecção por TB com doses semanais de rifapentina e isoniazida por três meses (3HP) sob a perspectiva do sistema público de saúde brasileiro. Os efeitos (TB evitada) e os custos de triagem e tratamento da infecção foram descontados em 5%. O custo-efetividade incremental por TB evitada foi calculado. Foram realizadas análises de sensibilidade unidirecionais e probabilísticas. O Brasil, um país de renda média-alta com alta carga de TB, possui um dos maiores sistemas públicos universais de saúde e oferece diagnóstico e tratamento gratuitos para TB. O TT é o atendimento padrão, o QFT-Plus está disponível para populações de risco muito elevado. Os três novos testes cutâneos estão em validação para eventual incorporação. Uma coorte hipotética de 10 mil profissionais de saúde, trabalhando em qualquer nível de atendimento, e resultados negativos de TT no ano anterior de ambos os sexos com um resultado de TT negativo na linha de base. Diaskintest, C-TST, Cy-TB e QFT-Plus têm maior especificidade. Os custos com o QFT-Plus foram maiores devido: equipamento, mão de obra e custo da testagem. O Diaskintest foi a estratégia de maior economia, seguido pelo Cy-TB para tratamento preventivo da TB com 3HP. No cenário brasileiro, Diaskintest e Cy-TB são os testes mais custo-efetivos para testagem sequencial de profissionais da saúde.

## Abstract (es)

Este estudio tuvo como objetivo analizar el costo-efectividad de tres pruebas cutáneas basadas en antígenos de tuberculosis (TB) (Diaskintest, C-TST, Cy-TB) y QFT-Plus para diagnosticar la infección por TB comparándolas con el estándar de atención actual, PPD Rt-23 prueba de la tuberculina (TT), entre profesionales de la salud en Brasil. Se trata de un modelo de Markov de transición de estado, que simula una cohorte de profesionales de la salud (cinco ciclos anuales) para pruebas y tratamiento de la infección por TB con dosis semanales de rifapentina e isoniazida durante tres meses (3HP) desde la perspectiva del sistema de salud pública brasileño. Los efectos (TB evitada) y los costos de detección y tratamiento de la infección se descontaron en un 5%. Se calculó el costo-efectividad incremental por TB evitada. Se realizaron análisis de sensibilidad unidireccionales y probabilísticos. Brasil, un país de ingresos medianos altos con una alta carga de TB, tiene uno de los mayores sistemas de salud pública universal y ofrece diagnóstico y tratamiento gratuitos para la TB. El TT es la atención estándar, QFT-Plus está disponible para poblaciones de muy alto riesgo. Las tres nuevas pruebas cutáneas se encuentran en proceso de validación para su eventual incorporación. Una cohorte hipotética de 10.000 profesionales de la salud, trabajando en cualquier nivel de atención, y resultados de TT negativos en el año anterior de ambos sexos con resultado de TT negativo en la línea de base. Diaskintest, C-TST, Cy-TB y QFT-Plus tienen mayor especificidad. Los costos con QFT-Plus fueron mayores debido a: equipo, mano de obra y costo de las pruebas. Diaskintest fue la estrategia más rentable, seguida de Cy-TB para tratamiento preventivo de la TB con 3HP. En el escenario brasileño, Diaskintest y Cy-TB son las pruebas más rentables para realizar pruebas secuenciales a los profesionales de la salud.

## Introduction

Brazil, an upper middle-income country, holds one of the largest universal public health systems, the Brazilian Unified National Health System (SUS, acronym in Portuguese), which covers over 150 million people who depend exclusively on its services. The Brazilian Ministry of Health frequently receives the highest budget of the Federal Government. This budget is dedicated to all health activities, thus incorporation of new technologies requires approval by an independent committee, which evaluates evidence on safety, effectiveness, and cost-effectiveness ^1^.

Brazil is one of the 30 highest tuberculosis (TB) burden countries ^2^. To achieve TB elimination, countries need to invest on TB preventive treatment ^3^. Since 2010, TB preventive treatment is recommended for all age contacts with a positive tuberculin skin test (TST) and a normal chest X-ray in Brazil, but uptake of TB preventive treatment has been slow ^4^. Healthcare workers are one of the priority contact populations, thus they should be tested for TB infection annually, and treated if conversion is detected and the chest X-ray is normal. Frequent losses occur in their cascade-of-care, particularly among healthcare workers with prior Bacilli Calmette-Guérin (BCG) vaccination, who are more likely to refuse TB preventive treatment ^5^. Pre-treatment losses, such as access to TB infection diagnostic tests, are also an important bottleneck ^6^ ^,^ ^7^.

Following the 2018 United Nations high-level meeting recommendations ^2^, some steps to scale up TB preventive treatment have been implemented in Brazil, including training of healthcare workers starting in 2018; implementation of a TB preventive treatment surveillance information system in the same year ^4^; incorporation of the QuantiFERON-TB Gold Plus (QFT-Plus) test (Qiagen; https://www.qiagen.com) for people living with HIV infection (PLWH), contacts aged 2-10, transplant candidates, and people using immunosuppressive drugs in 2021 ^8^; and incorporation of the 3HP regimen (three months of weekly doses of 900mg rifapentine and 900mg isoniazid) as the first choice for TB preventive treatment in 2022 ^9^.

The intermittent shortage of PPD Rt-23 (Statens Serum Institut; https://en.ssi.dk/) ^7^ and the lower specificity of TST in BCG-vaccinated populations ^10^ pose significant challenges, prompting the Brazilian National Tuberculosis Control Program (PNCT, acronym in Portuguese) to evaluate newer, more specific tests. QFT-Plus and TB antigen-based skin tests (TBST) use the more specific antigens, namely early secreted antigenic target 6 (ESAT-6) and culture filtrate protein 10 (CFP-10). Currently, three tuberculosis TBST are being validated in Brazil for eventual incorporation: Diaskintest (Generium Pharmaceutical; https://www.generium.ru/en/), C-TST (Zhifei Longcom Biologic Pharmacy Co.; https://en.zhifeishengwu.com), and Cy-TB (Serum Institute of India; https://www.seruminstitute.com/). QFT-Plus require laboratory facilities and is more costly than TST ^11^. Diaskintest was found to be cheaper and more effective than TST for PLWH in Brazil, but there is no data on cost-effectiveness of TBST in healthcare workers, the largest eligible population for TB preventive treatment ^12^. In this study, we analyzed the cost-effectiveness of TBST and QFT-Plus compared to TST for TB infection screening healthcare workers with a first negative TST test from the perspective of the SUS, over a 5-year time horizon.

## Methods

### Setting and current policies

The SUS provides, via the PNCT and municipal health departments, free-of-charge diagnosis and treatment for TB and TB infection. Investigation of TB infection in healthcare workers is recommended in Brazil as part of the worker’s pre-employment and annual health visits, regardless of the level of healthcare service ^13^. Healthcare workers with a first negative TST are invited to repeat the test in one to three weeks to assess the booster effect (induration size increment of 10mm). Individuals with a persistent negative TST then undergo a 1-step TST annually. TB preventive treatment is recommended when conversion (a 10mm increment over the latest induration size) occurs ^13^. Since March 2022, the TB preventive treatment regimen of choice is 3HP, except for children under two years of age, contacts over 50 years of age, those with liver disease, contacts of people with isoniazid- or rifampicin-monoresistant TB or intolerance to isoniazid or rifapentin ^9^. Prior to TB preventive treatment, for any indication, a chest radiograph is mandatory to rule out TB.

### Population, study perspective, time horizon, and discount rate

The study population consists of a hypothetical cohort of 10,000 Brazilian healthcare workers of both sexes with an average age of 35 years, working at any level of healthcare service and negative TST results in the previous year. The SUS perspective was adopted for this study ^1^. Each patient with positive bacilloscopy for *Mycobacterium tuberculosis* infects an average of 10 individuals per year 1 ^14^; about 1% to 5% of exposed individuals develop TB shortly after exposure (primary TB), and 10% to 30% become infected (TB infection) ^15^. Among those with TB infection, approximately 5% to 15% will develop the disease over their lifetime ^16^, with a higher risk in the first 2 to 5 years after infection ^17^. A time horizon of five years was considered, as individuals are at increased risk of developing TB in the first 2 to 5 years after TB infection ^17^. This study followed the recommendation of the Brazilian Ministry of Health, in which discount rates are standardized at 5% per year for effects and costs to increase the comparability of studies ^1^. Additionally, it suggests using different discount rates (0% and 10%) in sensitivity analysis to determine how much the arbitrary selection of the rate affected the study’s conclusion ^1^.

### Model structure

Given the increased risk of developing TB in the first 2 to 5 years after TB infection ^1^, a Markov decision-analytic model was developed to simulate a cohort of 10,000 healthcare workers over five annual cycles for TB preventive treatment with 3HP. Then, four strategies for TB infection detection were compared to the standard of care (TST), namely the QFT-Plus and three novel skin tests: Diaskintest, C-TST, and Cy-TB. The model simulated the natural history of TB infection, comparing the clinical outcomes and economic impacts of the newer diagnostic technologies to the traditional TST. All analyses were performed with TreeAge Pro Healthcare 2022 (https://www.treeage.com/). The health states considered were (1) no TB infection; (2) TB infection; (3) no TB infection, false positive test and not treated; (4) no TB infection, false positive test and treated; (5) TB infection and not tested; (6) TB infection, positive test and not treated; (7) TB infection, positive test and treated; (8) TB; (9) cured TB; and (10) death (Supplementary Material - Figure S1; https://cadernos.ensp.fiocruz.br/static//arquivo/suppl-e00178623_5325.pdf). The model estimated the number of TB cases that occurred in each of the strategies. The effectiveness measure was the number of TB cases avoided, estimated by subtracting the number of cases observed in the strategy with the reference test (TST) from those with the tests under evaluation (QFT-Plus and TBST). The number needed to misdiagnose was also estimated, defined as the number of patients who need to be tested in order for one to be misdiagnosed by the test (either false positive or false negative results), considering a 0.37 TB infection prevalence (95% confidence interval [95%CI]: 0.17; 0.36) ^18^ ^,^ ^19^. The incremental cost-effectiveness ratio (ICER) per TB case avoided was estimated by subtracting the number of cases observed in the strategy with the reference test (TST) from those with the tests under evaluation (QFT-Plus and TBST).

The probability of progression from TB infection to TB without TB preventive treatment was based on observed rates among healthcare workers for the first two years after infection, then it considered the probability for the general population ^20^.

### Testing procedures and interpretation

This study considered five strategies, with four being based on skin tests, three using the recombinant ESAT-6 and CFP-10 immunogens (Diaskintest, C-TST and Cy-TB), and one - the current standard of care - using the tuberculin PPD Rt-23 (TST). By replacing PPD with *M. tuberculosis* specific antigens, TBST combine the operational advantages of the TST with the specificity of interferon-gamma release assays (IGRA). The fifth strategy employs the QFT-Plus test, the only available IGRA in Brazil ^8^.

All skin tests involve the intradermal application of the antigen on the volar aspect of the forearm, following the Mantoux method in any healthcare facility (usually by a trained nurse) ^13^. The test result is read 48 or 72 hours after application. The cutoff point considered to define the result as positive was ≥ 5mm. If the reading is lost, no further skin tests is conducted and the healthcare workers should be retested after a 1-year interval.

QFT-Plus testing is conducted in the laboratory following the manufacturer’s instructions, using 1mL aliquots of whole blood, which can be obtained in the healthcare facility and transported to the laboratory or directly in the laboratory. If the result is indeterminate, the test is repeated once. Conversion is defined when a negative test turns positive ^13^. Only healthcare workers with a negative initial test undergo subsequent annual testing. When conversion of any TB infection test occurs, a chest radiograph is performed and if negative, TB preventive treatment is recommended ^13^.

### Model parameters

### Effectiveness data

Meta-analyses (Table 1) provided estimates for clinical, epidemiological and tests accuracy parameters ^21^ ^,^ ^22^ ^,^ ^23^ ^,^ ^24^ ^,^ ^25^ ^,^ ^26^ ^,^ ^27^ ^,^ ^28^ ^,^ ^29^ ^,^ ^30^. To estimate transition probabilities in the model, this study employed different types of measurement statistics reported in the literature using the method described by Gidwani & Russell ^31^ (Supplementary Material; https://cadernos.ensp.fiocruz.br/static//arquivo/suppl-e00178623_5325.pdf). Age-specific all-cause mortality rates were obtained from the 2022 Brazilian National Mortality Table ^32^.

**Table 1:** Model parameters: tests, treatment, status of infection, and outcomes.

Model parameters	Base-case	Range		Source
		Low	High	
Clinical and epidemiological parameters				
Incidence of TB infection in healthcare workers (based TST) *	0.17	0.09	0.24	^3^
Probability of not returning to TST treatment in healthcare workers	0.03	0.01	0.04	^45^
Probability of starting TB preventive treatment in healthcare workers **	0.84	0.67	1.00	^40^
Adherence to TB preventive treatment with 3HP ***	0.82	0.80	0.83	^28^
Efficacy of TB preventive treatment with 3HP ^#^	0.99	0.98	0.99	^28^
Probability of adverse events related to TB preventive treatment with 3HP ^##^	0.32	0.19	0.48	^29^
Probability of grade 3 or 4 adverse events related to TB preventive treatment with 3HP ^##^	0.03	0.02	0.06	^29^
Probability of adverse events that lead to permanent drug discontinuation related to TB preventive treatment with 3HP ^##^	0.08	0.06	0.10	^29^
Probability of death by adverse events related to TB preventive treatment with 3HP ^##^	0.0000004	0.0000	0.0010	^29^
Progression from TB infection to TB disease, no treatment (PPD-based TST ≥ 5mm) ^###^	0.007	0.000	0.015	^20^
Progression from TB infection to TB disease with complete TB preventive treatment with 3HP ^#^	0.001	0.0004	0.003	^28^
Test parameters				
TST sensitivity ^§^	0.77	0.71	0.82	^42^
TST specificity ^§§^	0.59	0.46	0.73	^42^
Diaskintest sensitivity (≥ 5mm) ^§§§^	0.91	0.81	0.96	^25^
Diaskintest specificity (< 5mm) ^†^	0.98	0.98	0.99	^26^
C-TST skin test sensitivity (≥ 5mm) ^††^	0.86	0.82	0.89	^25^
C-TST skin test specificity (< 5mm) ^†††^	0.98	0.98	0.99	^26^
Cy-TB skin test sensitivity (≥ 5mm) ^‡^	0.74	0.70	0.78	^25^
Cy-TB skin test specificity (< 5mm) ^‡‡^	0.98	0.94	0.99	^25^
QFT-Plus sensitivity ^‡‡‡^	0.91	0.87	0.94	^30^
QFT-Plus specificity ^∫^	0.97	0.95	0.98	^30^
Probability of indeterminate QFT-Plus ^∫∫^	0.01	0.005	0.04	^30^

3HP: three months of weekly doses of 900mg rifapentine and 900mg isoniazid; IGRA: interferon-gamma release assays; PPD: purified protein derivative; TB: tuberculosis; TST: tuberculin skin test.

* Incidence was defined as test conversion, including test definitions of IGRA and TST positivity and conversion for the studies included in this review. Data extracted from Figure 4 and Table S6 of the Apriani et al. ^3^;

** Data extracted from Table 2 of the Alsdurf et al. ^40^;

*** The study population included adults and children with high risk for developing TB disease. Data extracted from Analysis 4.4 of the Sharma et al. ^28^;

^#^ The study population included adults and children with high risk for developing TB disease. The relative risk was estimated by the authors for the risk of TB disease between the two groups under assessment. Data extracted from Analysis 4.1 of the Sharma et al. ^28^;

^##^ The study population included adults and children that started TB preventive treatment. Data extracted from Table 3;

^###^ Data extracted from Table 3 and Table S24 of the Campbell et al. ^20^;

^§^ Sensitivity was estimated among adults and children with TB disease. Data extracted from Figure 4 of the Pai et al. ^42^;

^§§^ Specificity was estimated among subjects with healthy low risk of TB infection with BCG vaccine scar. Summary measure estimated by bivariate random effects model. Data extracted from Table 1 and Supplementary Table 1 of the Pai et al. ^42^;

^§§§^ Sensitivity was estimated among adults and children with microbiologically confirmed TB disease. Data extracted from Supplementary Table S23 of the Krutikov et al. ^25^;

^†^ Specificity was estimated among healthy adults and children. Data extracted from Figure 4 and Table 1 of the Starshinova et al. ^26^;

^††^ Sensitivity was estimated among adults and children with microbiologically confirmed TB disease. Data extracted from Figure 3 of the Krutikov et al. ^25^;

^†††^ None of the systematic reviews evaluated estimated the specificity of the C-TST test. As an alternative, considering that both Diaskintest and C-TST are skin sensitivity tests and use the recombinant *Mycobacterium tuberculosis* antigens ESAT-6 and CFP-10, the specificity of C-TST was considered equal to that of Diaskintest;

^‡^ Sensitivity was estimated among adults and children with microbiologically confirmed TB disease. Data extracted from Figure 4 of the Krutikov et al. ^25^;

^‡‡^ Specificity was estimated among individuals without active tuberculosis in studies performed in tuberculosis low-incidence settings. Data extracted from Figure 5 of the Krutikov et al. ^25^;

^‡‡‡^ Sensitivity was estimated among patients with diagnosis of TB disease confirmed by molecular methods, or microbiological methods, or histopathology. Clinical diagnosis would be acceptable if methods of diagnosis were clearly described, and no TB infection test is incorporated in the definition. Data extracted from Table 2 and Supplementary Figure 2 of the Oh et al. ^30^;

^∫^ Specificity was estimated among adults with very low risk for TB infection. Data extracted from Table 2 and Supplementary Figure 3 of the Oh et al. ^30^;

^∫∫^ Data extracted from Table 4 of the Oh et al. ^30^ for studies that evaluated the sensitivity of the QFT-Plus.

### Costing data

Brazil boasts one of the largest universal public health systems (SUS), which provides coverage for over 150 million people who rely exclusively on its services. Given this, the national guideline for conducting economic evaluation studies recommends adopting the perspective of the SUS as the purchaser of services, thereby accounting for all costs covered by the public health system. The costs of medical visits, chest radiograph, sputum smear, blood work-up, aspartate aminotransferase (AST) and alanine aminotransferase (ALT) dosage, hospitalization, and death due to adverse events were obtained from the Brazilian Hospital Information System (SIH, acronym in Portuguese) ^33^. The costs for isoniazid (300mg/pill), rifapentine (150mg/pill), QFT-Plus (kit), and TST were informed by the PNCT (2024; personal communication). The cost of treating TB with directly observed therapy (DOT) were estimated by Steffen et al. ^34^. A micro-costing analysis for conducting diagnostic tests for TB infection was conducted and published elsewhere ^11^. Table 2 and Supplementary Material (https://cadernos.ensp.fiocruz.br/static//arquivo/suppl-e00178623_5325.pdf) detail the sources of costs estimates. All values were adjusted for inflation by the accumulated variation of the Brazilian Extended Consumer Price Index (IPCA, acronym in Portuguese) for the period and converted into U.S. dollars (USD) using the average annual rate according to Brazilian Central Bank (USD 1 = BRL 5.16) for 2022 ^35^, as described by Turner et al. ^36^ (Tables 2 and 3, and Supplementary Material; https://cadernos.ensp.fiocruz.br/static//arquivo/suppl-e00178623_5325.pdf).

**Table 2:** Model parameters: costs (in USD, 2022).

Model parameters	Cost estimates (in USD)			Source
	Base-case	Range		
		Low *	High **	
Drugs and exams				
Isoniazid (300mg/pill)	0.03	0.02	0.07	***
Rifapentine (150mg/pill)	0.26	0.13	0.53	***
Rifapentine and isoniazid (300mg/300mg/pill)	0.42	0.21	0.83	^37^
Blood count	1.78	0.89	3.57	^33^
Serum dosage AST	0.87	0.44	1.74	^33^
Serum dosage ALT	0.87	0.44	1.74	^33^
Medical visit	4.34	2.17	8.67	^33^
Chest radiograph	4.12	2.06	8.24	^33^
Sputum smear	1.82	0.91	3.64	^33^
TB infection diagnosis				
Initial medical visit (2 consultations)	8.67	4.34	17.35	^33^
Chest radiograph	4.12	2.06	8.24	^33^
Subtotal	12.79	6.40	25.59	
TB disease diagnosis				
Initial medical visit (2 consultations)	8.67	4.34	17.35	^33^
Chest radiograph	4.12	2.06	8.24	^33^
Sputum smear	1.82	0.91	3.64	^33^
Subtotal	14.62	7.31	29.23	
TB disease treatment with DOT ^#^	829.08	414.54	1,658.17	^34^
TB infection treatment with isoniazid (900mg/week) plus rifapentine (900mg/week) for 2 months (GDF)				
Rifapentine and isoniazid (900mg/900mg/week) (2 months/8 doses/24 pills)	10.00	5.00	20.00	^37^
Medical consultation (1 consultation)	4.34	2.17	8.67	^33^
Subtotal	14.34	7.17	28.67	
TB infection treatment with isoniazid (900mg/week) plus rifapentine (900mg/week) for 3 months (GDF)				
Rifapentine and isoniazid (900mg/900mg/week) (3 months/12 doses/36 pills)	15.00	7.50	30.00	^37^
Medical consultation (2 consultations)	8.67	4.34	17.35	^33^
Subtotal	23.67	11.84	47.35	
Adverse events				
Costs of grade 3 or 4 adverse events related to TB preventive treatment with 3HP	86.45	43.23	172.90	^33^
Adverse events follow-up				
Medical visit (3 consultations)	13.01	6.51	26.02	^33^
Blood count (2 exams)	3.57	1.78	7.13	^33^
Serum dosage AST (2 exams)	1.74	0.87	3.49	^33^
Serum dosage ALT (2 exams)	1.74	0.87	3.49	^33^
Subtotal	20.06	10.03	40.13	
Diagnostic tests				
QFT Plus				
Human resources ^##^	1.73	0.87	3.46	^33^
QFT-Plus test kit ^###^	17.68	8.84	35.36	***
Consumables ^§^	1.40	0.70	2.80	^11^
Equipment ^§§^	0.83	0.41	1.65	^11^
Subtotal	21.64	10.82	43.27	
TST PPD Rt-23 (2UT/1.5mL)				
Human resources ^##^	1.64	0.82	3.28	^11^
Consumables ^§§§^	1.01	0.51	2.02	^11^
Equipment ^†^	0.04	0.02	0.08	^11^
PPD Rt-23 (2UT/1.5mL)	0.93	0.47	1.86	***
Subtotal	3.62	1.81	7.24	
Diaskintest				
Human resources ^##^	1.64	0.82	3.28	^11^
Consumables ^§§§^	1.01	0.51	2.02	^11^
Equipment ^†^	0.04	0.02	0.08	^11^
Diaskintest	1.43	0.72	2.86	^††^
Subtotal	4.12	2.06	8.24	
C-TST				
Human resources ^##^	1.64	0.82	3.28	^11^
Consumables ^§§§^	1.01	0.51	2.02	^11^
Equipment ^†^	0.04	0.02	0.08	^11^
C-TST skin test	6.09	3.05	12.18	^12^
Subtotal	8.78	4.39	17.56	
Cy-TB				
Human resources ^##^	1.64	0.82	3.28	^11^
Consumables ^§§§^	1.01	0.51	2.02	^11^
Equipment ^†^	0.04	0.02	0.08	^11^
Cy-TB skin test	1.00	0.50	2.00	^††^
Subtotal	3.69	1.84	7.38	
Cost of death for adverse events related to TB preventive treatment with 3HP	441.20	220.60	882.40	^11^
Discount rate	0.05	0.00	0.10	^1^

3HP: three months of weekly doses of 900mg rifapentine and 900mg isoniazid; ALT: alanine aminotransferase; AST: aspartate aminotransferase; DOT: directly observed therapy; GDF: Global Drug Facility; PPD: purified protein derivative; TB: tuberculosis; TBST: tuberculosis antigen-based skin tests; TST: tuberculin skin test.

* The lower limit was 50% of the cost estimate value;

** The upper limit was double the cost estimate value;

*** The costs for isoniazid (300mg/pill), rifapentine (150mg/pill), QFT-Plus (kit), and TST were identified from personal communication with the Brazilian National Tuberculosis Control Program;

^#^ DOT costs based on five weekly visits during the intensive phase (first 2 months) and twice weekly during the continuation phase (remaining 4 months);

^##^ Nursing staff time (for TBST and TST only), laboratory technician time (for QFT-Plus only);

^###^ QFT-Plus test kit included blood collection tubes, microplates, and reagents for the enzyme-linked immunosorbent assay;

^§^ Gloves, needles, tourniquet, cotton, alcohol, box for syringes, Eppendorf, cryotube, color-coded insert (red and blue), DNA Free Pyrogen (200μL - sterile and with filter), and D1000 Diamond Tipack 100-1000μL (sterile and with filter);

^§§^ Incubator, centrifuge, microplate washer, microplate reader, computer, and printer;

^§§§^ Gloves, cotton, alcohol, syringes with needles, box for syringes, thermal box, and ice bag;

^†^ Fridge, thermometer with alarm, and millimeter ruler;

^††^ The costs of the Cy-TB test and Diaskintest were identified from personal communication with the manufacturers.

The complete TB preventive treatment costs included 12 weekly doses of rifapentine (900mg) and isoniazid (900mg) and two medical monitoring visits; 3HP was self-administered ^9^. Brazil does not acquire consumables from the Global Drug Facility (GDF); thus, information were obtained from the Brazilian Ministry of Health, and the GDF value considered the combined weekly dose of 3HP in the sensitivity analysis ^37^.

Partial treatment costs for TB infection were considered for those who developed severe adverse events and did not complete TB preventive treatment. The incomplete TB preventive treatment included costs with two months of weekly doses of rifapentine (900mg) and isoniazid (900mg) and one medical consultation.

Cases of severe adverse events with 3HP incurred in hospitalization costs, valued by the code *Treatment of Complications of Surgical or Clinical Procedures* from the SIH table, with a 35% increase in values related to professional care in hospitals considered as type II urgency ^33^.

In cases of adverse events that evolved to death, costs equivalent to two daily hospitalizations to the intensive care unit were included ^33^. For those who survived, the monitoring costs of severe adverse events were also included, encompassing three doctor visits, two blood count tests, and two tests for AST and ALT.

### Sensitivity analyses

One-way sensitivity analysis was conducted on all model inputs, using the 95%CI range of transition probabilities. For the cost parameters, the lower limit of the range was half the price and the upper limit, twice the price ^1^. A probabilistic sensitivity analysis was also performed in a Monte Carlo simulation with 10,000 iterations (Table 3). It was assumed that the uncertainty in clinical probabilities and accuracies followed a beta distribution, with the standard deviation (SD) estimated based on data extracted from systematic literature reviews (Supplementary Material; https://cadernos.ensp.fiocruz.br/static//arquivo/suppl-e00178623_5325.pdf). A gamma distribution was assumed for costs, with the SD equal to 10% of the original value. The generated distributions of costs, TB cases avoiding diagnosis of TB infection and its preventive treatment, and ICER are shown using median and 95% uncertainty ranges (UR).

**Table 3:** General model parameters and distributions.

Model parameters	Deterministic mean	PSA probability distribution	SD	Source
Clinical and epidemiological parameters				
Incidence of TB infection in healthcare workers treated with TST	0.17	Beta	0.008	^3^
Probability of not returning to TST treatment in healthcare workers	0.03	Beta	0.006	^43^
Probability of starting TB preventive treatment in healthcare workers	0.84	Beta	0.012	^21^
Adherence to TB preventive treatment with 3HP	0.82	Beta	0.006	^28^
Efficacy of TB preventive treatment with 3HP	0.99	Beta	0.0006	^28^
Probability of adverse events related to TB preventive treatment with 3HP	0.32	Beta	0.005	^29^
Probability of grade 3 or 4 adverse events related to TB preventive treatment with 3HP	0.04	Beta	0.002	^29^
Probability of adverse events that lead to permanent drug discontinuation related to TB preventive treatment with 3HP	0.080	Beta	0.002	^29^
Probability of death by adverse events related to TB preventive treatment with 3HP	0.0000004	Beta	0.000005	^29^
Progression from TB infection to TB disease, no treatment (based TST ≥ 5mm)	0.007	Beta	0.004	^20^
Progression from TB infection to TB disease with complete TB preventive treatment with 3HP	0.001	Beta	0.001	^28^
TST sensitivity (≥ 5mm)	0.77	Beta	0.012	^41^
TST specificity (< 5mm)	0.59	Beta	0.020	^41^
Diaskintest sensitivity (≥ 5mm)	0.91	Beta	0.034	^25^
Diaskintest specificity (< 5mm)	0.98	Beta	0.00007	^26^
C-TST test sensitivity (≥ 5mm)	0.86	Beta	0.016	^25^
C-TST test specificity (< 5mm)	0.98	Beta	0.00007	^26^
QFT-Plus sensitivity	0.91	Beta	0.011	^30^
QFT-Plus specificity	0.97	Beta	0.009	^30^
Probability of indeterminate QFT-Plus	0.01	Beta	0.003	^30^
Cy-TB test sensitivity (≥ 5mm)	0.74	Beta	0.020	^25^
Cy-TB test specificity (< 5mm)	0.98	Beta	0.007	^25^
Cost estimates (in USD, 2022)				
TB infection diagnosis	12.79	Gamma	2.56	^33^
TB disease diagnosis	14.62	Gamma	2.92	^33^
TB disease treatment with DOT *	829.08	Gamma	165.82	^34^
TB preventive treatment with rifapentine (900mg/week) plus isoniazid (900mg/week) for 2 months	17.83	Gamma	3.57	^11^ **
TB preventive treatment with 3HP	28.91	Gamma	5.78	^11^ **
TB preventive treatment with rifapentine (900mg/week) plus isoniazid (900mg/week) for 2 months (GDF)	14.34	Gamma	2.87	^11, 40^
TB preventive treatment with 3HP (GDF)	23.67	Gamma	4.73	^11, 40^
Treatment for grade 3 or 4 adverse events related to TB preventive treatment with 3HP	86.45	Gamma	17.29	^33^
Adverse events follow-up	20.06	Gamma	4.01	^33^
QFT Plus test ***	17.68	Gamma	3.54	**
TST PPD Rt-23 (2UT/1.5mL)	0.93	Gamma	0.19	**
Diaskintest	1.43	Gamma	0.29	^#^
C-TST skin test	6.09	Gamma	1.22	^12^
Cy-TB skin test	1.00	Gamma	0.20	^#^
Human resources for TST and TBST ^##^	1.64	Gamma	0.33	^11^
Consumables for TST and TBST ^###^	1.01	Gamma	0.20	^11^
Equipment for TST and TBST ^§^	0.04	Gamma	0.01	^11^
Human resources for QFT-Plus ^##^	1.73	Gamma	0.35	^11^
Consumables for QFT-Plus ^§§^	1.40	Gamma	0.28	^11^
Equipment for QFT-Plus ^§§§^	0.83	Gamma	0.17	^11^
Cost of death for adverse events	441.20	Gamma	88.24	^33^
Discount rate	0.05	Triangular	-	^1^

3HP: three months of weekly doses of 900mg rifapentine and 900mg isoniazid; DOT: directly observed therapy; GDF: Global Drug Facility; PPD: purified protein derivative; PSA: probabilistic sensitivity analysis; SD: standard deviation; TB: tuberculosis; TBST: tuberculosis antigen-based skin tests; TST: tuberculin skin test.

* DOT costs based on 5 weekly visits during the intensive phase (first 2 months) and twice weekly during the continuation phase (remaining 4 months);

** The costs for isoniazid (300mg/pill), rifapentine (150mg/pill), QFT-Plus (kit), and TST were identified from personal communication with the Brazilian National Tuberculosis Control Program;

*** QFT-Plus test kit included blood collection tubes, microplates, and reagents for the enzyme-linked immunosorbent assay;

^#^ The costs of the Cy-TB test and Diaskintest were identified from personal communication with manufacturers;

^##^ Nursing staff time (for TBST and TST only), laboratory technician time (for QFT-Plus only);

^###^ Gloves, cotton, alcohol, syringes with needles, box for syringes, thermal box, and ice bag;

^§^ Fridge, thermometer with alarm, and millimeter ruler;

^§§^ Gloves, needles, tourniquet, cotton, alcohol, box for syringes, Eppendorf, cryotube, color-coded insert (red and blue), DNA Free Pyrogen (200μL - sterile and with filter), and D1000 Diamond Tipack 100-1000μL (sterile and with filter);

^§§§^ Incubator, centrifuge, microplate washer, microplate reader, computer, and printer.

### Model assumptions

The cohort undergoes standard procedures for healthcare workers, as prescribed by the Brazilian national guidelines. Several simplifying assumptions were incorporated into the model: (1) all healthcare workers were asymptomatic and without TB; (2) no one was HIV-infected; (3) healthcare workers with a positive test result underwent chest radiography and physical examination to exclude TB; (4) chest radiography and symptom screening are 100% accurate in ruling out TB; (5) individuals with a second indeterminate result on the QFT-Plus test had the same risk of developing TB as those with a negative result; (6) nonadherence to treatment due to adverse events occurred in the first two months and conferred no partial protection; (7) all individuals with TB accepted treatment; (8) patients who completed TB treatment were considered cured; (9) all TB and TB infection cases are sensitive to antituberculosis drugs; (10) people who did not adhere to TB preventive treatment did not develop adverse events; (11) the death of an healthcare workers from other causes had no financial impact on the Brazilian Ministry of Health; and (12) our assessment considered the stability of newer recombinant reagents to be comparable to that of PPD Rt-23.

### Willingness-to-pay threshold

In Brazil, the government-established threshold for cost-effectiveness is USD 7,752 per quality-adjusted life-year (QALY) gained ^38^. Due to the lack of a threshold reference for case avoidance in Brazil, the QALY threshold was adopted as a reference, which has been the metric used for the incorporation of technologies into the SUS. To assess the cost-effectiveness of the intervention, this threshold was employed for each TB case prevented. Then, a cost-effectiveness acceptability curve was constructed, which illustrates the probability of the intervention being deemed cost-effective across a range of willingness-to-pay threshold values.

### Price threshold analysis

Price threshold analysis was conducted to determine the maximum price at which the strategy incorporating QFT-Plus would be considered cost-effective. This analysis was carried out probabilistically, enabling the assessment of uncertainties in the model parameters and their influence on the cost-effectiveness of the intervention.

This cost-effectiveness report followed the *Consolidated Health Economic Evaluation Reporting Standards 2022* (CHEERS 2022) statement ^39^.

## Results

Table 4 presents the projected costs and health outcomes of simulated strategies. The SUS costs for screening 10,000 healthcare workers over a 5-year period, including screening and treatment costs for both TB infection using weekly 3HP doses and TB treatment with DOT, were: USD 298,236 (95%UR: 251,196; 353,603) for Diaskintest; USD 286,640 (95%UR: 241,310; 340,003) for Cy-TB test; USD 434,984 (95%UR: 357,541; 525,148) for C-TST test; USD 334,619 (95%UR: 273,173; 409,566) for TST; and USD 801,727 (95%UR: 620,926; 1,028,999) for QFT-Plus. Compared to TST, the Diaskintest and Cy-TB strategies were the most cost saving for TB infection diagnosis (USD 7,239 and USD 62,388 per TB case averted, respectively) (Supplementary Material - Figure S2 and Table S4; https://cadernos.ensp.fiocruz.br/static//arquivo/suppl-e00178623_5325.pdf). Despite QFT-Plus demonstrating slightly higher effectiveness compared to Diaskintest and Cy-TB, the incremental cost of using QFT-Plus was USD 84,038 per TB case averted (Table 4).

**Table 4:** Strategy rankings - all referencing common baseline (in USD, 2022).

Strategy	Cost per 10,000 healthcare workers	95%UR		Incremental cost per 10,000 healthcare workers	TB disease cases	95%UR		TB disease cases averted	ICER	95%UR	
		Low	High			Low	High			Low	High
TST	334,619	273,173	409,566	-	16	14	17	-	-	-	-
Diaskintest	298,236	251,196	353,603	-36,383	11	9	13	5	-7,239	-4,229	-12,885
C-TST	434,984	357,541	525,148	100,365	12	11	13	4	25,917	22,385	29,663
Cy-TB	286,640	241,310	340,003	-47,979	15	13	17	1	-62,388	-31,709	-153,995
QFT-Plus	801,727	620,926	1,028,999	467,108	10	9	11	6	84,038	66,875	105,846

ICER: incremental cost-effectiveness ratio; TB: tuberculosis; TST: tuberculin skin test; UR: uncertainty ranges.

With regards to number needed to misdiagnose, 29, 92, 155, 191, and 217 tests are necessary for 10 healthcare workers to be misdiagnosed by the TST, Cy-TB, C-TST, QFT-Plus, and Diaskintest, respectively.

Monte Carlo simulations showed that, at a willingness to pay threshold of USD 7,752 per TB case averted and considering all screening strategies simultaneously, the Diaskintest showed the highest net benefit in 92.9%. The Cy-TB strategy presented the highest net benefit in 7.1%. Cost-effectiveness acceptability curve, illustrating the proportion of simulations in which each strategy holds the highest net benefit at different willingness to pay thresholds is provided in Supplementary Material (Figure S3; https://cadernos.ensp.fiocruz.br/static//arquivo/suppl-e00178623_5325.pdf).

One-way sensitivity analyses demonstrated that the incremental cost-effectiveness of TB infection screening strategies among healthcare workers was most sensitive to estimated TB infection incidence in healthcare workers (TST), the cost of TB preventive treatment, the cost of excluding TB, and the cost of Diaskintest (Supplementary Material - Table S4 and Figure S4; https://cadernos.ensp.fiocruz.br/static//arquivo/suppl-e00178623_5325.pdf). The cost-effectiveness estimates for Cy-TB strategy was also sensitive to TST specificity (Supplementary Material - Table S4 and Figure S4; https://cadernos.ensp.fiocruz.br/static//arquivo/suppl-e00178623_5325.pdf).

Considering the GDF value for the combined weekly dose of 3HP, a cost reduction is observed for all screening strategies: USD 281,689 for Diaskintest; USD 270,768 for Cy-TB; USD 418,605 for C-TST test; USD 308,507 for TST; and USD 784,573 for QFT-Plus. When compared to TST, assuming a higher probability of progression from TB infection to TB in the first two years after infection (0.015) in healthcare workers, the Diaskintest strategy remains the most cost saving for TB infection diagnosis, with savings of USD 5,244 per TB case averted.

Price threshold analysis, at a willingness-to-pay threshold of USD 7,752 per TB case averted, showed that for the QFT-Plus strategy to be more cost-effective when compared to Diaskintest, it should cost USD 0.16. On the other hand, for the screening strategy with QFT-Plus to be more cost-effective when compared to that of Cy-TB, the QFT-Plus test should cost USD 0.91.

## Discussion

In this cost-effectiveness model, we compared the Diaskintest screening strategy with the TST for Brazilian healthcare workers. The Diaskintest test was the most cost saving, followed by the Cy-TB test. The QFT-Plus test was more costly with slightly higher effectiveness. Diaskintest has also been shown to be cost saving in Brazilian PLWH ^13^. The main reason for these findings is the costs of tests. All ESAT-6/CFP-10-based tests, whether in vivo (TBST) or ex vivo (QFT-Plus), exhibit similar accuracy ^25^. Consequently, their cost-effectiveness is based primarily on their costs. The QFT-Plus (and other World Health Organization [WHO]-approved IGRA tests) requires equipment, laboratory infrastructure, sample transportation from health facilities to the laboratory, and labor-intensive processes. Consumables (mainly the test kits) also contribute to the cost. In contrast, while the tuberculin unit is expensive, the test requires less labor (approximately 6.5 minutes total in low-and middle-income countries) ^40^ and can be conducted in any clinic with a refrigerator for storing vials and properly trained personnel.

The WHO recommends TB infection tests for high-risk populations, such as PLWH and household contacts with bacteriologically confirmed pulmonary TB (regardless of age or HIV status) ^41^. In Brazil, TB infection tests are mandatory for most individuals undergoing TB preventive treatment ^4^. Our findings support the Brazilian recommendation and suggest that Diaskintest or another specific test with similar costs should be incorporated. However, there is currently more evidence on the safety and accuracy of the Cy-TB test, and decisions to incorporate new technologies into the health system follows a multicriteria logic. As for QFT-Plus to be more cost-effective in this population, it should cost less than USD 1.

While both Diaskintest and Cy-TB exhibited cost-saving advantages over alternative interventions, their overall savings are contingent upon various factors, including final costs, which are subject to fluctuations in exchange rates and the optimization of reagent supply chains. Factors such as storage requirements, dosage per vial, and reagent stability post-opening significantly influence final test costs and, consequently, their cost-effectiveness. Moreover, integrating these tests into programs targeting TB preventive treatment scale up could stimulate local innovation and foster competition to meet regional demand. Recurrent consumable stock-outs could also be overcome by the availability of different tests. An additional benefit compared to IGRAs is the absence of implementation costs, given their long-standing use as standard practice in Brazil and the current efforts to expand skin test competence in healthcare workers (ExpandTPT project, 2024; personal communication). Finally, skin tests are provided at the point of care, which enables a wider availability of these tests in rural and other remote areas.

An universal limitation of economic analyses of TB infection detection and treatment is related to the sensitivity of tests. Since no golden standard exists for TB infection diagnosis, these economic analyses are based on studies performed in populations with TB, in whom TST, TBST, and IGRA tests, which are based on immune response, may show lower sensitivity ^42^. Previous studies were also sensitive to TB infection incidence, a parameter with a high degree of uncertainty among healthcare workers ^3^. We used a large range of TB infection incidence (9%-24%) in our sensitivity analysis, and there was no impact in the final conclusion.

This study shows several limitations. Firstly, decision analyses inherently depend on the quality and accuracy of their base-case modeling parameter values. We did not consider drug-resistant TB strains due to their modest prevalence in Brazil and the current recommendation not to treat contacts of persons with drug-resistant TB ^43^. Moreover, we did not consider the entire TB spectrum (incipient and sub-clinical disease) ^44^, as no commercially available tests or treatment implications exist for other TB status. Additionally, our model is based on numerous assumptions. We tested the potential impact of major assumptions using sensitivity analyses, while others were assumed as fixed. For instance, our study did not model HIV infection, TB treatment dropout rates, or skin test booster effects. We also did not address the cost-effectiveness of baseline screening at hiring, which is necessary to identify workers who would benefit from subsequent tests and to interpret the results, regardless of the specific test and frequency adopted. We did not consider costs for repeated skin tests in cases of lost readings, but we estimated the rate of non-return at 3% ^45^. Finally, due to the lack of a threshold reference for case avoidance in Brazil, we opted to use the QALY threshold as a reference, which has been the metric used for the incorporation of technologies into the SUS, notwithstanding the limitations of this comparison ^1^ ^,^ ^38^.

Despite these limitations, this cost-effectiveness analysis can contribute to decision-making in Brazil and serve as a reference for economic evaluations in other countries. The findings suggest that alternatives to the QFT-Plus approach may be worth considering, as these new tests could potentially facilitate the scale-up of TB prevention programs and contribute to global TB control efforts. Importantly, these alternatives may not require venipuncture or the use of costly laboratory facilities.
