# Supplementary material for: TB antigen-based skin tests and QFT-Plus for Mycobacterium tuberculosis infection diagnosis in Brazilian healthcare workers: a cost-effectiveness analysis
Source: Cad Saude Publica. 2025 Feb 24;41(1):e00178623. doi: 10.1590/0102-311XEN178623 (PMC11863634; doi:10.1590/0102-311XEN178623)
Supplement: Supplementary file 1 [file 1678-4464-csp-41-02-EN178623-s.pdf]

Summary

1. Simplified diagram of clinical transition states of Markov model 4

2. Transition probabilities 7

3. Standard deviation and confidence interval 9

4. Number needed to misdiagnose 9

5. Costing data 10

6. Supplementary results 16

References 24

List of supplementary tables

Supplementary table 1 -	Conversion of the cost parameters identified in the Brazilian Hospital Information System (SIH-SUS) table to 2022 US dollar (US$ ([rate US$1 = R$5.16]).	9
Supplementary table 2 -	Correction for inflation and conversion of cost parameters estimated by Loureiro et al., 2019.	11
Supplementary table 3 -	Correction for inflation and conversion of cost parameters estimated by Steffen et al., 2010.	12
Supplementary table 4	Summary of univariate sensitivity analyses for tuberculosis prevention treatment with 3HP.	14

List of supplementary figures

Supplementary figure 1	Simplified diagram of clinical transition states of Markov model. Arrows indicate the direction in which a healthcare worker moves from one state to another each year. The probability of death, defined according to the Brazilian life table, was applied to all health states considered.	4
Supplementary figure 2 –	Cost-effectiveness acceptability curves (CEACs) of the different diagnostic strategies for tuberculosis infection for TBI treatment regime with weekly doses of rifapentine (900 mg) and isoniazid (900 mg).	13
Supplementary figure 3 -	Tornado diagram for one-way sensitivity analysis of incremental cost-effectiveness ratio using US$ per avoided tuberculosis case for TBI treatment regime with weekly doses of rifapentine (900 mg) and isoniazid (900 mg).	15

1. CHEERS 2022 Checklist

Topic	No.	Item	Location where item is reported
**Title**			
	1	Identify the study as an economic evaluation and specify the interventions being compared.	Title page
**Abstract**			
	2	Provide a structured summary that highlights context, key methods, results, and alternative analyses.	Abstract
**Introduction**			
**Background and objectives**	3	Give the context for the study, the study question, and its practical relevance for decision making in policy or practice.	1 and 2
**Methods**			
**Health economic analysis plan**	4	Indicate whether a health economic analysis plan was developed and where available.	No
**Study population**	5	Describe characteristics of the study population (such as age range, demographics, socioeconomic, or clinical characteristics).	3
**Setting and location**	6	Provide relevant contextual information that may influence findings.	2 and 3
**Comparators**	7	Describe the interventions or strategies being compared and why chosen.	5 and 6
**Perspective**	8	State the perspective(s) adopted by the study and why chosen.	3 and 4
**Time horizon**	9	State the time horizon for the study and why appropriate.	3 and 4
**Discount rate**	10	Report the discount rate(s) and reason chosen.	3 and 4
**Selection of outcomes**	11	Describe what outcomes were used as the measure(s) of benefit(s) and harm(s).	4 and 5
**Measurement of outcomes**	12	Describe how outcomes used to capture benefit(s) and harm(s) were measured.	4

Topic	No.	Item	Location where item is reported
**Valuation of outcomes**	13	Describe the population and methods used to measure and value outcomes.	4
**Measurement and valuation of resources and costs**	14	Describe how costs were valued.	6, 7 and Supplementary material
**Currency, price date, and conversion**	15	Report the dates of the estimated resource quantities and unit costs, plus the currency and year of conversion.	6, 7 and Supplementary material
**Rationale and description of model**	16	If modelling is used, describe in detail and why used. Report if the model is publicly available and where it can be accessed.	4 and title page
**Analytics and assumptions**	17	Describe any methods for analysing or statistically transforming data, any extrapolation methods, and approaches for validating any model used.	6, 7 and Supplementary material
**Characterising heterogeneity**	18	Describe any methods used for estimating how the results of the study vary for subgroups.	Not applicable
**Characterising distributional effects**	19	Describe how impacts are distributed across different individuals or adjustments made to reflect priority populations.	5
**Characterising uncertainty**	20	Describe methods to characterise any sources of uncertainty in the analysis.	7 and 8
**Approach to engagement with patients and others affected by the study**	21	Describe any approaches to engage patients or service recipients, the general public, communities, or stakeholders (such as clinicians or payers) in the design of the study.	Not applicable
**Results**			
**Study parameters**	22	Report all analytic inputs (such as values, ranges, references) including uncertainty or distributional assumptions.	Table 1, table 2 and table 3
**Summary of main results**	23	Report the mean values for the main categories of costs and outcomes of interest and summarise them in the most appropriate overall measure.	Table 1, table 2 and table 3
**Effect of uncertainty**	24	Describe how uncertainty about analytic judgments, inputs, or projections affect findings. Report the effect of choice of discount rate and time horizon, if applicable.	Table 5

Topic	No.	Item	Location where item is reported
**Effect of engagement with patients and others affected by the study**	25	Report on any difference patient/service recipient, general public, community, or stakeholder involvement made to the approach or findings of the study	Not applicable
**Discussion**			
**Study findings, limitations, generalisability, and current knowledge**	26	Report key findings, limitations, ethical or equity considerations not captured, and how these could affect patients, policy, or practice.	11 - 14
**Other relevant information**			
**Source of funding**	27	Describe how the study was funded and any role of the funder in the identification, design, conduct, and reporting of the analysis	Title page
**Conflicts of interest**	28	Report authors conflicts of interest according to journal or International Committee of Medical Journal Editors requirements.	Title page

From: Husereau et al. (2022).[1]

2. Simplified diagram of clinical transition states of Markov model

Supplementary figure 1 - Simplified diagram of clinical transition states of Markov model. Arrows indicate the direction in which a healthcare worker moves from one state to another each year. The probability of death, defined according to the Brazilian life table, was applied to all health states considered.

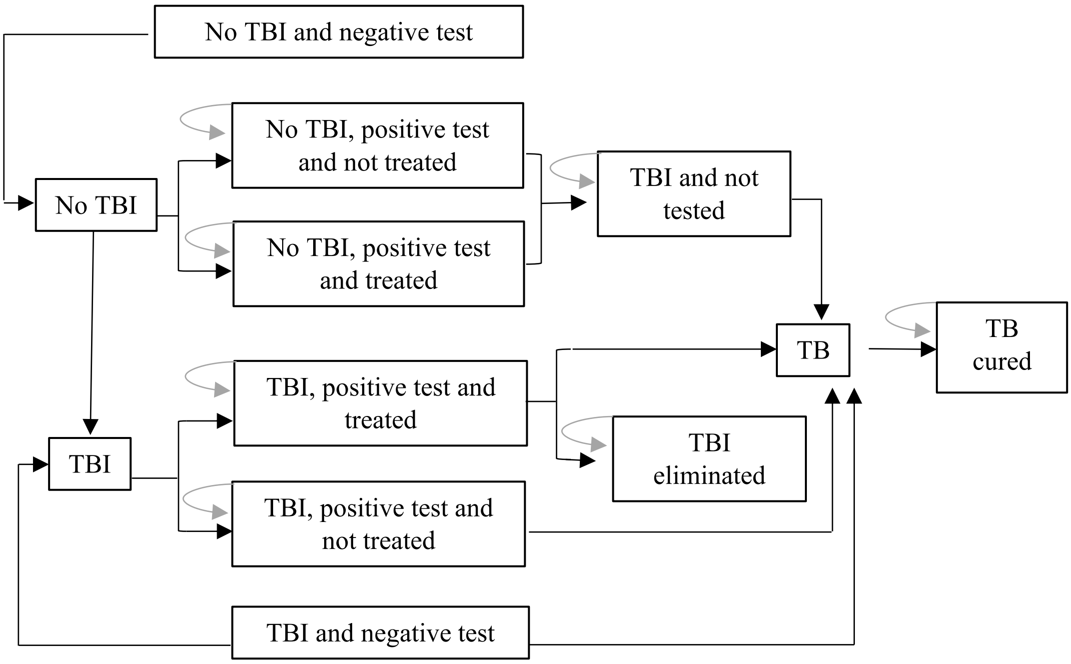

Abbreviations: TBI – tuberculosis infection; TB – tuberculosis disease.

3. Transition probabilities

A set of health states or events and the probabilities of transition from one state to another during a pre-specified time interval are fundamental for the construction of decision models. In general, transition probabilities are not available in the literature in a format directly suitable for application in these models, which makes it necessary to use mathematical procedures to estimate them based on evidence available in the literature, but in other types of measures. summaries.[2]

Gidwani and Russell (2020) presents practical guidance on transforming various types of information published in journals, or available online from government and other sources, into transition probabilities for use in state-transition models, including cost-effectiveness models.[2]

Also according to Gidwani and Russell (2020),[2] when the summary measure available is the relative risk or the risk ratio (RR) (Equation 1):

$$RR = p1/p0 \tag{1}$$

where p1 is the probability of the event under assessment in those exposed to the intervention and p0 is the probability of the event under assessment in those not exposed, the following Equation 2 for converting the RR into probability of transition can be applied:

$$p1 = RR * p0 \tag{2}$$

Summary measure is the odds ratio (OR), Gidwani and Russell (2020)[2] propose (Equation 3):

$$RR = \frac{OR}{\left(1 - p0 + (p0 * OR)\right)} \tag{3}$$

that, after replacing the RR variable in Equation 1 with Equation 3, we convert the summary measures of the OR type into transition probability according to Equation 4:

$$p1 = \frac{OR*p0}{(1-p0+(p0*OR))} \tag{4}$$

In the references whose summary measures were available referring to the event of interest (e.g. number of TB cases identified in patients who received the treatments under comparison rather than the effectiveness of the treatment; or number of TBI cases

who abandoned the treatment of according to comparison groups rather than the treatment adherence), the probability of transition was estimated after calculating the OR (Equation 5) or RR (Equation 1) for the measure of interest according to the summary measure used by the reference .

$$OR = (\frac{p1}{1 - p1})/(\frac{p0}{1 - p0}) \quad (5)$$

4. Standard deviation and confidence interval

The standard deviation (σ) of the sample proportion (p) was estimated with Equation 6:

$$\sigma = \frac{\sqrt{p\,(1 - p)}}{n} \quad (6)$$

Finally, the 95% confidence intervals (CI) of the transition probabilities were estimated for the sample proportion with Equation 7:

$$95\%\ CI = p\ \pm 1{,}96 * \ \sigma \quad (7)$$

5. Number needed to misdiagnose

The number needed to misdiagnose (NNM) is the number of patients who need to be tested in order for one to be misdiagnosed by the test.[3] The index can be calculated as follows (Equation 8):

$$NNM = \frac{1}{\left(1 - Sp - Pr(Se * Sp)\right)} \quad (8)$$

where Pr represents pretest probability (prevalence of the disease), Sp is the specificity and Se sensibility.

6. Costing data

Estimated costs in previous years were adjusted for inflation for the period. The inflation correction factor was calculated by the ratio between the IPCA (*Índice Nacional de Preços ao Consumidor Amplo)* index number in December 2022 and the IPCA index number in December of the year in which the cost was estimated.[4] Then, the costs were adjusted for inflation and converted into US dollars according to Turner et al. (2019).[5]

The costs for medical visit; chest radiograph; sputum smear; blood count; serum dosage the AST (aspartate aminotransferase) and ALT (alanine aminotransferase); hospitalization for severe adverse events (code "Treatment of complications of surgical or clinical procedures"); and daily hospitalizations to the intensive care unit adult (ICU III) were identified in the Brazilian Hospital Information System (SIH-SUS) table (http://sigtap.datasus.gov.br/tabela-unificada/app/sec/procedimento/publicados/consultar) with last updated in 2008. The costs were adjusted for inflation for the period (between December 2008 and December 2022, correction factor = 2.23), then converted the Brazilian reais (R$) to 2022 US dollars using the annual average rate (US$ [rate US$1 = R$5.16]) (Supplementary table S1).

The costs of supplies (consumption of reagents and materials, as examination gloves, needles syringes, tourniquet, cotton, alcohol, box for syringes), and of equipment

(fridge for TBST, thermometer with alarm, millimeter ruler for reading TBST, incubator, centrifuge, microplate washer, microplate reader, computer, printer) and human resources (nurses and laboratory technicians) as estimated by Loureiro et al. (2019) between 2013-2014.[6] The costs were first converted to 2014 Brazilian reais (US$ [rate US$1 = R$ 2.35]), then adjusted for inflation for the period [between December 2013 (IPCA= 3815.39) and December 2022 (IPCA= 6474.09), correction factor = 1.69] and, finally, converted to 2022 US dollars (US$ [rate US$1 = R$5.16]) (Supplementary table 2).[5]

Supplementary table S1 - Conversion of the cost parameters identified in the Brazilian Hospital Information System (SIH-SUS) table to 2022 US dollar (US$ ([rate US$1 = R$5.16]).

Code[1]	Procedure	Cost in R$ (2008)	Cost in R$ (2022)[2]	Cost in US$ (2022)
02.04.03.015-3	Chest radiograph	9.50	21.26	4.12
02.02.01.064-3	Serum dosage GOT	2.01	4.50	0.87
02.02.01.065-1	Serum dosage GPT	2.01	4.50	0.87
02.02.08.004-8	Sputum smear	4.20	9.40	1.82
03.01.01.007-2	Medical visit	10.00	22.38	4.34
02.02.02.038-0	Blood count	4.11	9.20	1.78
03.08.04.001-5	Treatment of complications of surgical or clinical procedures[3]	199.33	446.09	86.45
08.02.01.009-1	Daily hospitalizations to the intensive care unit adult[4]	508.63	1138.29	220.60

Abbreviations: Brazilian Hospital Information System (SIH); AST - aspartate aminotransferase ; ALT - alanine aminotransferase; R$ - Brazilian reais; US$ US dollars; SUS - *Sistema Único de Saúde*.

(1) Procedure code in Brazilian Hospital Information System (SIH-SUS) table.

(2) The period considered by correction the inflation was between December 2008 (IPCA = 2892.86) and December 2022 (IPCA = 6474.09). The correction factor used was 2.23.

(3) Code includes International Statistical Classification (ICT) T887 (Unspecified adverse drug effect).

(4) In cases of severe adverse events that evolved to death, costs equivalent to two daily hospitalizations to the intensive care unit adult (ICU III) were included.

The cost of treating TB with directly observed therapy (DOT) were estimated by Steffen et al. (2010).[7]

The objective of the study was to analyze the costs of care TB patients undergoing treatment in facilities using the DOT and facilities providing only self-administered therapy in Rio de Janeiro State (RJ), Brazil. In addition, the extra costs of treatment supervision to the patient and the health system were estimated to calculate the incremental cost-effectiveness ratio (ICER) of the DOT strategy per completed treatment. Costs per completed treatment were US$ 194 for patients and U$ 189 for the health system in self-administered therapy (SAT) facilities, compared to US$ 336 and US$ 726 in DOT facilities.[7]

The costs estimated by Steffen et al. (2010) were converted to 2008 Brazilian reais (US$ [rate US$1 = R$ 1.80]), then adjusted for inflation for the period (between December 2008 (IPCA = 2892.86) and December 2022, (IPCA = 6474.09) (correction factor = 2.23) and, finally, converted to 2022 US dollars (US $ [rate US$1 = R$5.16]) (Supplementary table 3).[7]

Costs for C-TST® were identified by Steffen et al. (2020), converted to 2020 Brazilian reais (US$ [rate US$1 = R$ 4.50]), adjusted for inflation (between December 2020 and December 2022, correction factor = 1.16) and then converted to 2022 US dollars using the annual average rate (US$ [rate US$1 = R$5.16]).[8]

Supplementary table S2 – Correction for inflation and conversion of cost parameters estimated by Loureiro et al. (2019).

Cost parameters	Cost in US$ (2013 - 2014)	Cost in R$ (2014)[1]	Cost in R$ (2022)[2]	Cost in US$ (2022)[3]
Consumables for skin tests	1.31	3.08	5.22	1.01
Equipment for skin tests	0.05	0.12	0.20	0.04
Human resources for skin tests	2.12	4.98	8.45	1.64
Consumables for QFT-Plus®	1.81	4.25	7.22	1.40
Equipment for QFT-Plus®	1.07	2.51	4.27	0.83
Human resources for QFT-Plus®	2.24	5.26	8.93	1.73

Abbreviations: IPCA - *Índice Nacional de Preços ao Consumidor Amplo*; QFT-Plus® - QuantiFERON-TB Gold Plus; R$ - Brazilian reais; US$ - US dollars.

(1) US$ 1 = R$ 2.35 (in 2014).

(2) The period considered for correction for inflation was between December 2013 (IPCA = 3815.39) and December 2022 (IPCA = 6474.09). The correction factor used was 1.69.

(3) US$ 1 = R$ 5.16 (in 2022).

The costs for isoniazid (300 mg/ pill); rifapentine (150 mg/ pill); QFT-Plus® (kit); and TST were identified from personal communication with National Tuberculosis Program (NTP) in 2022.

Supplementary table S3 – Correction for inflation and conversion of cost parameters estimated by Steffen et al. (2010).

**Cost parameters**	**Cost in US$ (2008)**	**Cost in R$ (2008)[1]**	**Cost in R$ (2022)[2]**	**Cost in US$ (2022)[3]**
TB treatment with DOT[4]	1062.00	1911.60	4278.07	829.08

Abbreviations: DOT - directly observed therapy ; IPCA - *Índice Nacional de Preços ao Consumidor Amplo*; TB - tuberculosis disease; R$ - Brazilian reais; US$ - US dollars.

(1) US$ 1 = R$ 1.80 (in 2008).

(2) The period considered for correction for inflation was between December 2008 (IPCA = 2892.86) and December 2022 (IPCA = 6474.09). The correction factor used was 2.23.

(3) US$ 1 = R$ 5.16 (in 2022).

(4) Costs per completed treatment was US$ 336 for patients and US$ 726 in DOT facilities (Steffen et al., 2010).[7]

7. Supplementary results

Supplementary figure 2 - (a–d) Incremental cost-effectiveness scatter plot of (a) QFT-Plus® vs. TST; (b) C-TST® vs. TST; (c) Cy-TB® vs. TST; (d) Diaskintest® vs TST. Costs in 2022 US$ and effectiveness in avoided tuberculosis cases for TBI treatment regime with weekly doses of rifapentine (900 mg) and isoniazid (900 mg).

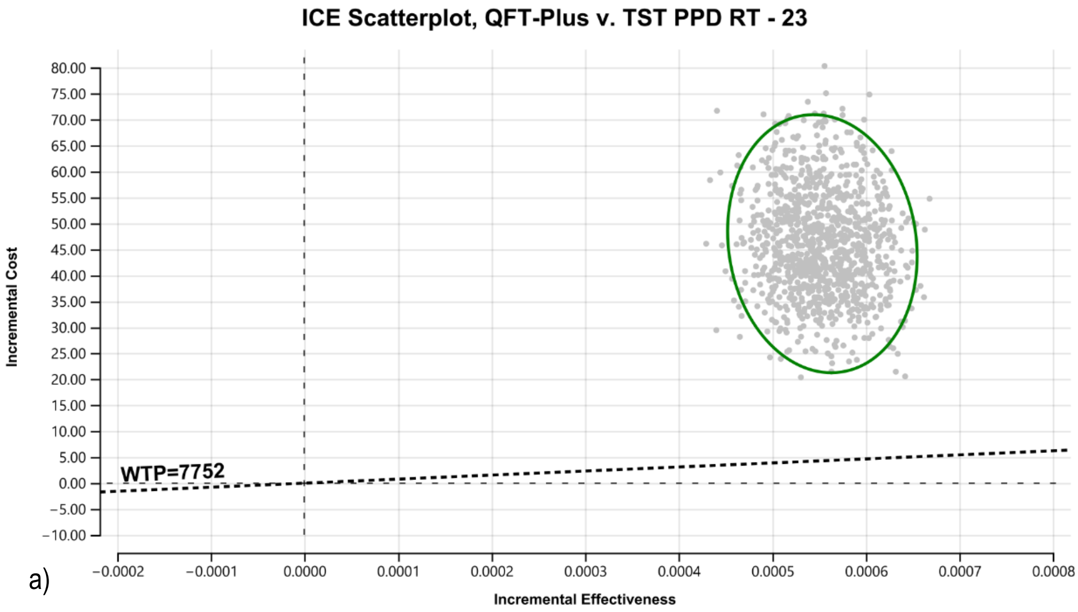

a)

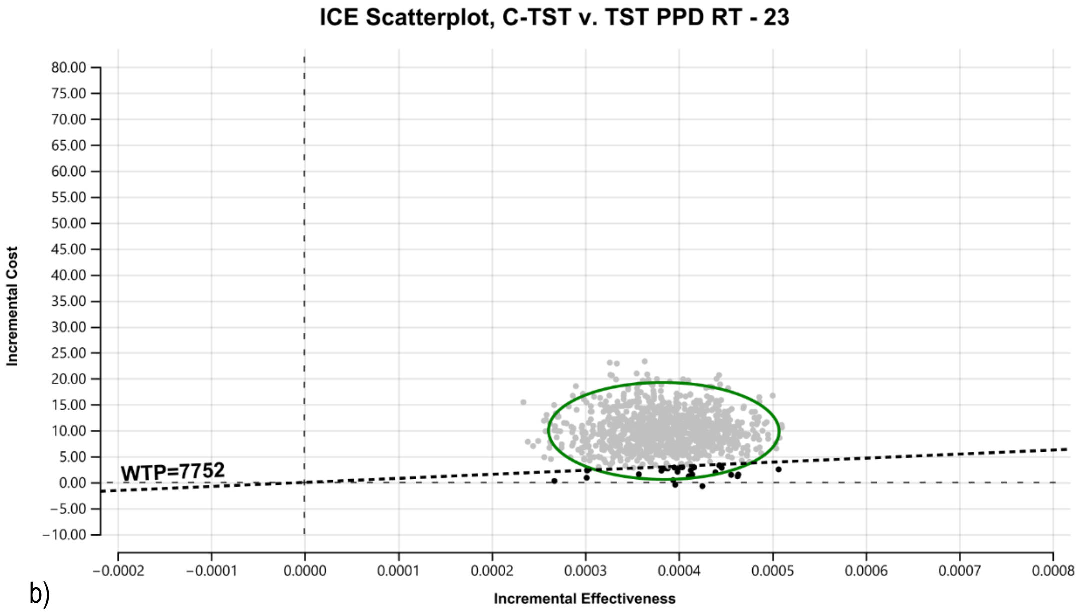

b)

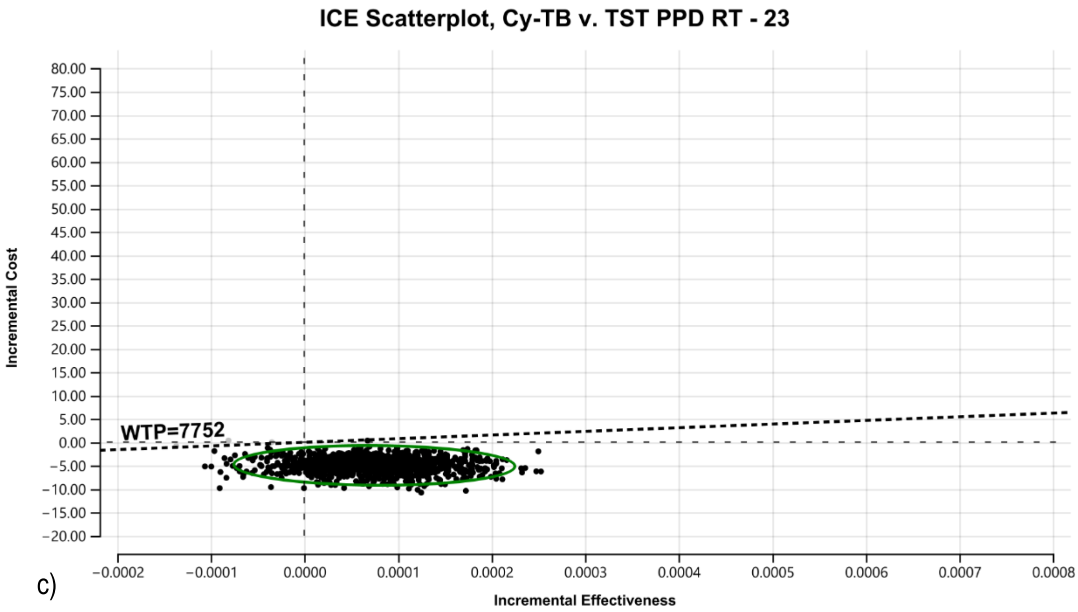

c)

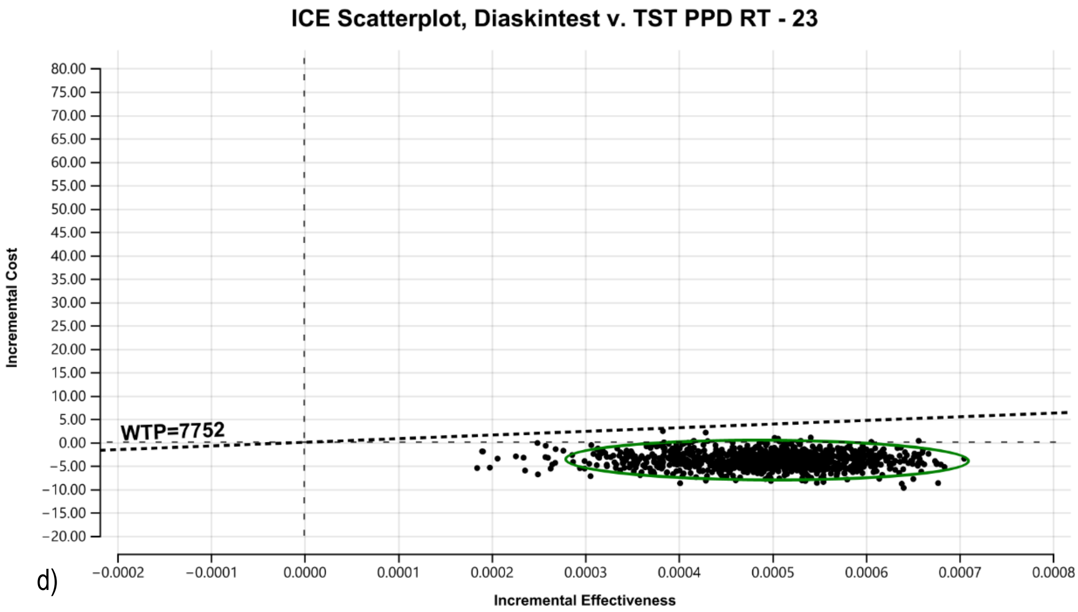

d)

The graphs show the scatter plot of the resulting incremental cost-effectiveness ratio of 10,000 Monte Carlo simulations for the different strategies compared with TST based PPD. The diagonal line represents the willingness to pay threshold of US$ 7,752. Values on the right lower quadrant are cost saving (less costly and more effective). The X-axis is scaled from − 0.001 to 0.001 avoided tuberculosis cases and Y-axis from − 100 to 100 US$.

Abbreviations: PPD Rt 23 - purified protein derivative; QFT-Plus® - QuantiFERON-TB Gold Plus; TST - tuberculin skin test; WTP - willingness-to-pay threshold.

Supplementary figure 3 - Cost-effectiveness acceptability curves (CEACs) of the different diagnostic strategies for TBI and TPT regime with weekly doses of rifapentine (900 mg) and isoniazid (900 mg).

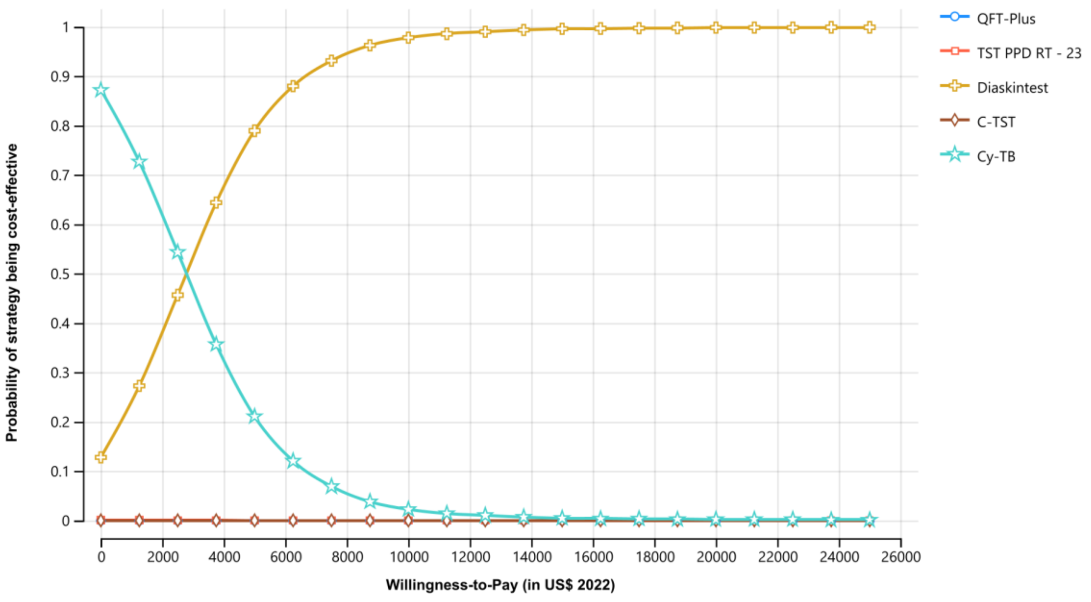

Cost effectiveness acceptability curves using the net-monetary benefit approach (10,000 Monte Carlo iterations) represent the probability (y-axis) that each strategy is more cost effective compared at the range of willingness to pay thresholds (US$ per avoided tuberculosis case) on the x-axis. The curve is generated by repeating the procedure for various thresholds, with the threshold on x-axis and the probability to be cost effective on y-axis. Acceptability curves are presented here considering direct costs only.

Abbreviations: QFT-Plus®- QuantiFERON TB Gold Plus; PPD- purified protein derivative; TBI– tuberculosis infection; TPT - tuberculosis preventive treatment; TST- tuberculin skin test, US$- US dollars.

Supplementary table 4 - Summary of univariate sensitivity analyses for tuberculosis prevention treatment with 3HP.

TST versus Diaskintest®						
**Variable description**	**Variable low**	**Base**	**Variable high**	**Impact**	**Low**	**High**
TBI incidence in HCWs	0.09	0.17	0.24	Increase	-22708.49	-2972.11
Cost TPT with 3HP	14.45	28.91	57.81	Decrease	-16315.69	-2697.27
Cost of excluded TB in TBI diagnosis	6.4	12.79	25.59	Decrease	-15821.96	-2953.98
Cost of Diaskintest®	0.72	1.43	2.86	Increase	-11327.29	995.64
Diaskintest® sensitivity (≥ 5 mm)	0.81	0.91	0.96	Increase	-13441.34	-6044.25
Human resources costs for TST and TBST	0.82	1.64	3.28	Increase	-9566.49	-2583.55
Discount rate annual	0	0.05	0.1	Decrease	-10158.35	-4480.37
**TST versus Cy-TB® skin test**						
TST specificity (< 5 mm)	0.46	0.59	0.73	Decrease	-177037.34	-39408.41
TBI incidence in HCWs	0.09	0.17	0.24	Increase	-165168.96	-31242.85
Cost TPT with 3HP	14.45	28.91	57.81	Decrease	-125901.18	-30609.49
Cost of excluded TB in TBI diagnosis	6.4	12.79	25.59	Decrease	-122446.43	-32405.77
Discount rate annual	0	0.05	0.1	Decrease	-117695.91	-32369.50
Probability of starting TBI treatment between HCWs	0.67	0.84	1	Increase	-109612.04	-50205.04
Cost of Cy-TB® test	0.5	1	2	Increase	-81943.79	-23276.54

Abbreviations: 3HP - 3 months of weekly doses of rifapentine (900 mg / week) and isoniazid (900 mg / week); TB - tuberculosis disease; TBI - tuberculosis infection; TBST - tuberculosis antigen-based skin tests; TST - tuberculin skin test.

Supplementary figure 4 - Tornado diagram for one-way sensitivity analysis of incremental cost-effectiveness ratio using US$ per avoided tuberculosis case for TPT with weekly doses of rifapentine (900 mg) and isoniazid (900 mg).

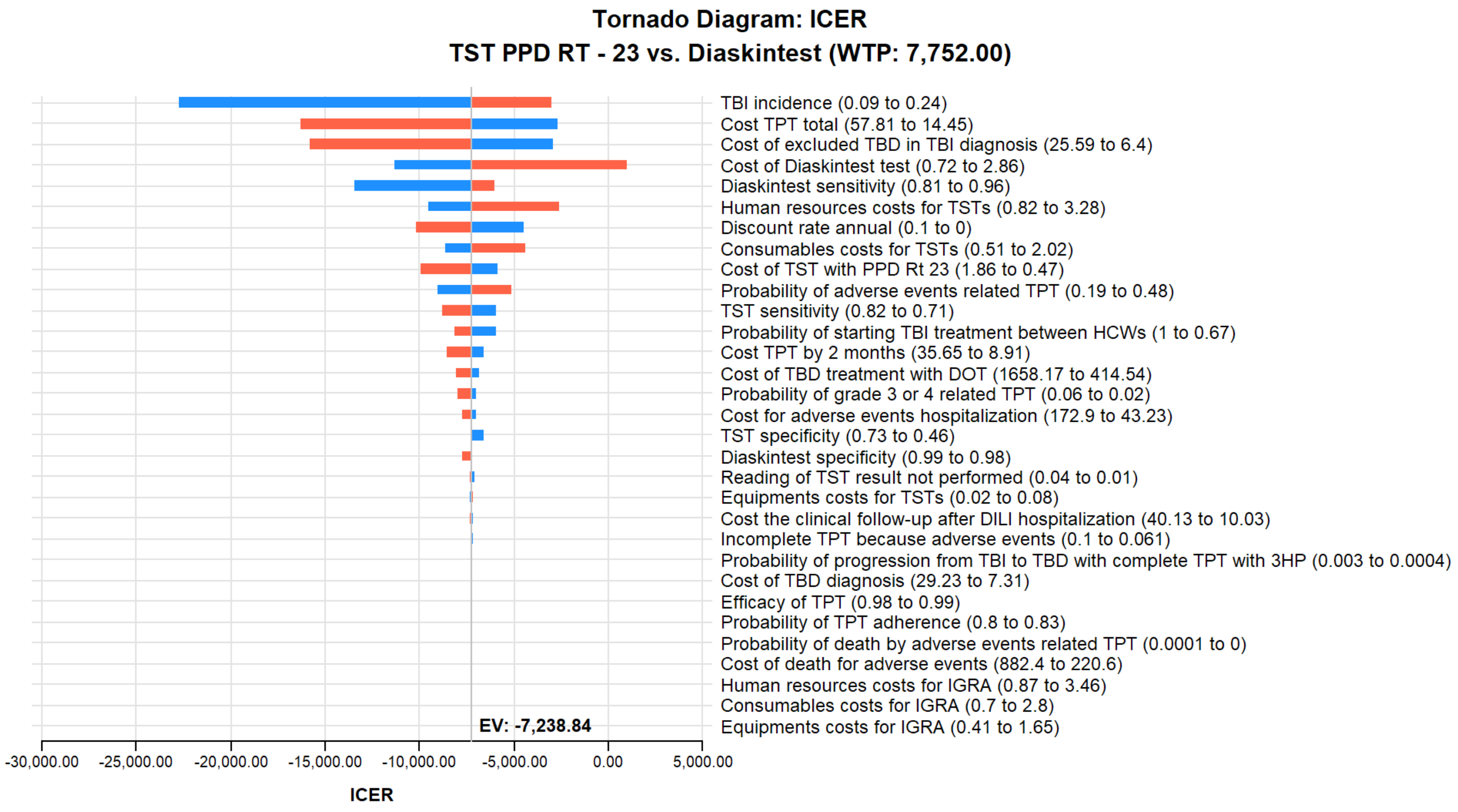

a)

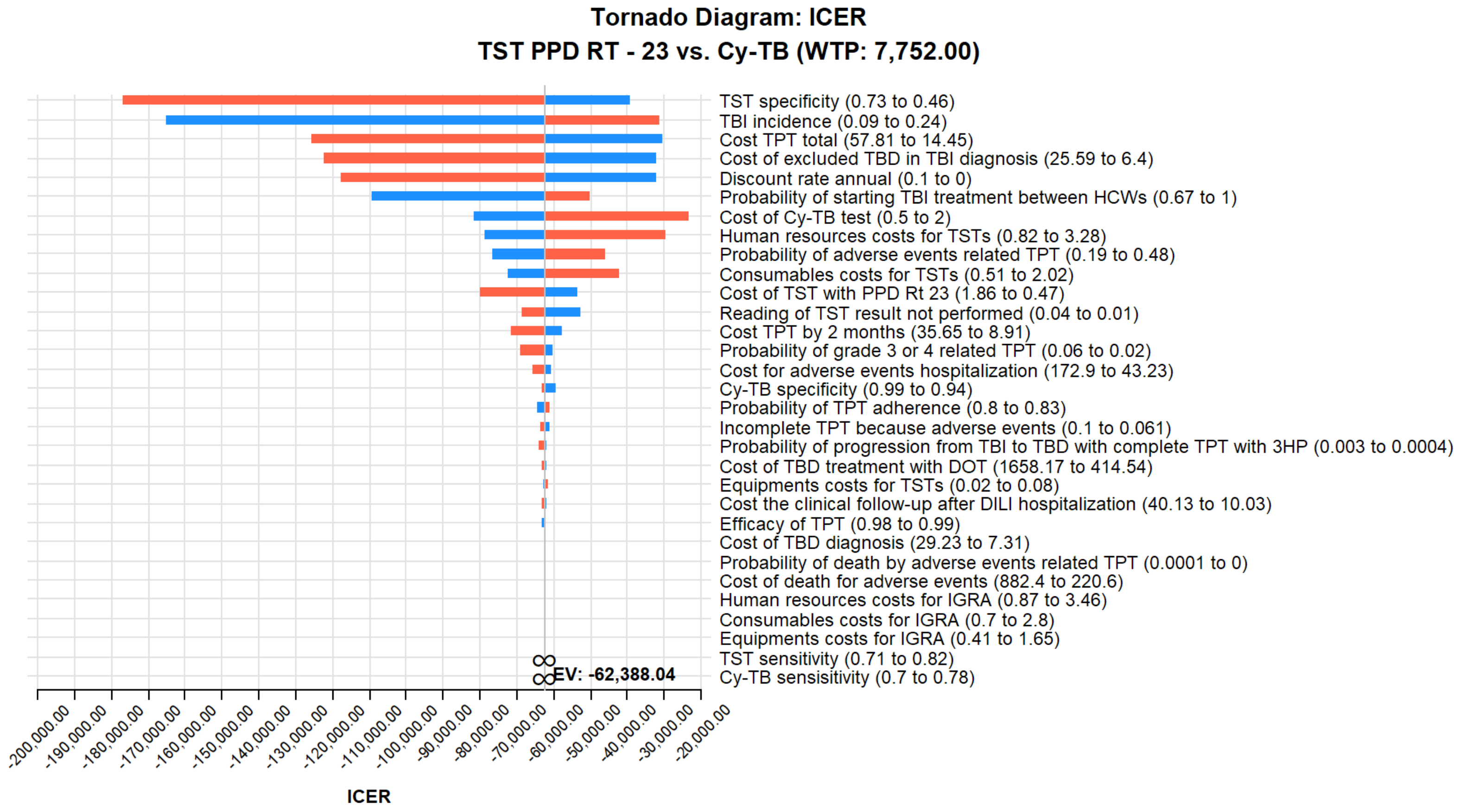

b)

Horizontal bars show the variation in incremental cost-effectiveness ratio (ICER; in US$ per avoided tuberculosis cases) with variation in the value of the parameter. (a) TST with PPD Rt 23 versus Diaskintest®; (b) TST with PPD Rt 23 versus Cy-TB®.

Abbreviations: HCW– healthcare worker; ICER- Incremental cost-effectiveness ratio; IGRA- interferon-gamma release assays; PPD- purified protein derivative; TST- tuberculin skin test; TBD– tuberculosis disease; TPT- tuberculosis prevention treatment; TBI- tuberculosis infection.

References

1. Husereau D, Drummond M, Augustovski F, de Bekker-Grob E, Briggs AH, Carswell C, et al. Consolidated Health Economic Evaluation Reporting Standards (CHEERS) 2022 Explanation and Elaboration: A Report of the ISPOR CHEERS II Good Practices Task Force. Value Heal [Internet]. 2022;25(1):10–31. Available from: https://doi.org/10.1016/j.jval.2021.10.008
2. Gidwani R, Russell LB. Estimating Transition Probabilities from Published Evidence: A Tutorial for Decision Modelers. Pharmacoeconomics [Internet]. 2020;38(11):1153–64. Available from: https://doi.org/10.1007/s40273-020-00937-z
3. Habibzadeh F, Yadollahie M. Number needed to misdiagnose: A measure of diagnostic test effectiveness. Vol. 24, Epidemiology. 2013. p. 170.
4. IBGE - INSTITUTO BRASILEIRO DE GEOGRAFIA E ESTATÍSTICA. Inflação [Internet]. 2021 [cited 2022 Jan 5]. Available from: https://www.ibge.gov.br/explica/inflacao.php
5. Turner HC, Lauer JA, Tran BX, Teerawattananon Y, Jit M. Adjusting for Inflation and Currency Changes Within Health Economic Studies. Value Heal [Internet]. 2019;22(9):1026–32. Available from: https://doi.org/10.1016/j.jval.2019.03.021
6. Loureiro RB, Maciel ELN, Caetano R, Peres RL, Fregona G, Golub JE, et al. Cost-effectiveness of QuantiFERON-TB Gold In-Tube versus tuberculin skin test for diagnosis and treatment of Latent Tuberculosis Infection in primary health care workers in Brazil. PLoS One. 2019;14(11):e0225197.
7. Steffen R, Menzies D, Oxlade O, Pinto M, de Castro AZ, Monteiro P, et al.

Patients’ costs and cost-effectiveness of tuberculosis treatment in dots and non-dots facilities in Rio de Janeiro, Brazil. PLoS One. 2010;5(11).

8. Steffen RE, Pinto M, Kritski A, Trajman A. Cost-effectiveness of newer technologies for the diagnosis of Mycobacterium tuberculosis infection in Brazilian people living with HIV. Vol. 10, Scientific Reports. 2020.
